# Supplementary material for: “My home is (now) at peace”: Evaluating the relevance, acceptability and potential scalability of a guided self-help intervention for male refugees in Uganda
Source: PLOS Ment Health. 2025 Mar 17;2(3):e0000079. doi: 10.1371/journal.pmen.0000079 (PMC12798302; doi:10.1371/journal.pmen.0000079)
Supplement: Attachment — Submitted filename: Response to reviewers.docx [file pmen.0000079.s002.docx]

**Response to reviewers:** “My home is (now) at peace”: Evaluating the relevance, acceptability and potential scalability of a guided self-help intervention for male refugees in Uganda

The authors wish to thank the associate editor and reviewers for their helpful reviews of the manuscript. Please find below point by point responses to the feedback received. Our responses are numbered and provided in *italics* for ease of reading. Any quotes from the revised paper are additionally *underlined*.

**Associate Editor:**

***Response:*** *Thank you for the reviewers’ feedback and the opportunity to revise our manuscript.*

**Response to Reviewer #1:**

**R1:1** Overall, this was a very interesting and relevant paper. However, there is an opportunity for the authors to further distinguish their findings by contextualizing the study in a cultural context earlier on. Despite all of the participants being of South Sudanese descent, no specific mention of cultural nuances was made until the discussion section. More information on cultural dynamics, cultural differences between refugees and the host community, and displacement context will help establish a better understanding from readers who may be unfamiliar with the adversities faced by this specific refugee population in this particular host country.

***Response:*** Thank you for highlighting this. We agree that providing cultural context earlier in the manuscript would enhance the reader’s understanding. We have revised the **introduction** to include a discussion of South Sudanese cultural norms, particularly around masculinity, substance use, and coping mechanisms:

*This study focuses on male South Sudanese refugees living in northern Uganda, where displacement has created significant challenges. Traditionally, South Sudanese men are viewed as family providers and protectors, but unemployment and reliance on humanitarian aid have disrupted these roles, leading to a loss of identity, heightened psychological distress, and increased substance misuse (18). The cultural expectation of masculinity intensifies these struggles, as many men face the burden of being unable to fulfill their traditional roles (18). Additionally, cultural differences between refugees and the Ugandan host community, along with stigma surrounding mental health, exacerbate feelings of isolation and contribute to worsening outcomes (18).* (page 4, line 82 - 90)

Introduction
**R1.2** Line 51-54: gender disparities in psychological distress and substance misuse are mentioned without a description of the specific factors unique to male refugees that worsen these outcomes.

***Response:*** *Thank you for your reflections and feedback. We have integrated our reflections on gender disparities together with the reviewers comment* above (page 4, line 82 - 90)

**R1.3** Line 56-59: the prevalence of alcohol misuse among male refugees is reported, but could use more context as to why this is a coping mechanism often used by this population.

***Response:*** *Thank you for the feedback. We had added the following in the* ***introduction****:*

*In many cases, psychological distress—such as anxiety, depression, and trauma—leads to alcohol misuse as a coping mechanism, providing temporary relief. However, this misuse worsens mental health issues, creating a vicious cycle of emotional distress and impaired functioning (5). This bidirectional relationship is particularly evident in refugee populations, where displacement and the loss of social support increase vulnerability to both conditions (6).* (page 3, line 53 – 59)

*Alcohol is often used as a coping mechanism to relieve stress from adapting to new environments and to manage mental health conditions such as traumatic stress, anxiety, and depression (8). Among displaced populations, alcohol misuse is particularly common, offering temporary relief from these psychological challenges (9). Displacement can leave men feeling helpless and socially isolated, which often drives them to alcohol as an escape (10). However, while it may offer short-term relief, alcohol misuse worsens mental health issues and reinforces harmful behaviours, making it a maladaptive strategy with long-term detrimental effects.* (page 3- 4, line 63 – 69)

**R1.4** Line 60-61: the relationship between alcohol misuse and mental health needs to be more clearly established. The statistics leading up to this claim only report the prevalence of each concern independently.

***Response:*** *We appreciate this point and we have integrated our response together with the revieweres comment above (R1.3)* (page 3, line 53 – 59)

**R1.5** Line 63-68: the connection between gaps in mental health resources and access to actual intervention content is unclear.

***Response:*** *Thank you for the feedback. We have added the following in the* ***introduction****:*

*Despite the clear need for interventions, access to mental health services remains limited in humanitarian settings. Most settings lack adequate mental health facilities, trained professionals, and specialized services to meet the complex needs of male refugees (11). Scalable interventions are essential in these resource-constrained contexts. Task shifting, where non-specialists deliver mental health care, offers a promising solution (12). By enabling briefly trained individuals to provide mental health support, task shifting bridges the gap in services, making care more accessible and sustainable in under-resourced areas (13).* (page 4, line 74 – 81)

**R1.6** Line 72-75: stigma and discrimination are noted only in the introduction and not circled back in the results/discussion.

***Response:*** *Thank you for the feedback*. We have added the following in the discussion:

*While discussing alcohol misuse and psychological distress, participants also highlighted the stigma and discrimination they faced within their communities and from their families. For many, excessive drinking was associated with social isolation, either self-imposed or as a result of being isolated by family and community members. Participants shared that drinking alcohol was perceived negatively in society, leading them to hide their behaviour or drink in isolation. This stigma compounded their mental health challenges and created additional barriers to seeking support. The combined intervention played a key role among participants to overcome this social stigma, rebuild their relationships, and reintegrate into their communities.* (page, 32, line 637 – 644)

Methods
**R1.7** Why was the study conducted in the two refugee settlements in northern Uganda? The introduction mentioned limited evidence on alcohol misuse and mental health challenges among refugees in low and middle-income countries. Was there any other reason besides the host country's economic status?

***Response:*** *The study was conducted in two refugee settlements in northern Uganda for several reasons beyond the host country's economic status. Northern Uganda hosts a large population of South Sudanese refugees, making it an ideal location to examine the unique mental health and alcohol misuse challenges faced by this group. Additionally, Uganda’s progressive refugee policy, which integrates refugees into host communities, provided a valuable context for studying social dynamics and their impact on mental health. SH+ has also been tested in this setting before (*[*Tol et al., 2020*](https://www.thelancet.com/journals/langlo/article/PIIS2214-109X(19)30504-2/fulltext)*), offering a relevant foundation for evaluating the combined intervention in a similar context with a focus on scalability and real-world applicability.*

**R1.8** Was there any specific reasoning behind the inclusion criteria related to the participants? Or was this just a matter of a majority of refugees in these settlements participating in this program being South Sudanese males?

***Response:*** *The inclusion criteria were specifically chosen to focus on South Sudanese males because they represent the majority of refugees in these settlements and are disproportionately affected by both psychological distress and alcohol misuse. This population's cultural and social context, particularly around disrupted gender roles, made them a key focus for assessing the effectiveness of the intervention. Therefore, the criteria were aligned with the study’s objective to address these specific challenges in a targeted way.*

**R1.9** 181 participants met the inclusion criteria but only 18 participated in the process evaluations (28 participants including family and facilitators). Was this drop in participants solely due to availability and willingness? Were all 181 participants presented with the opportunity to participate or was there a cap due to resource limitations?

***Response:*** *All participants were offered the opportunity to participate in the process evaluations. However, only those who consented to be interviewed, and who were available and willing, were included in the evaluation. The drop in participants was primarily due to availability and willingness, as refugees are highly mobile and often engaged in other livelihood activities that can limit their availability for interviews.*

**R1.10** Line 162-163: note that scores greater than or equal to 5 were classified as moderate psychological distress based on “previous studies with South Sudanese refugees” but only cite one empirical study. Based on the original instrument, scores of 8-12 indicated moderate distress.

***Response:*** *Thank you for the observation. The classification of scores greater than or equal to 5 as moderate psychological distress was informed by a combination of the original instrument and adjustments made based on empirical findings from a study with South Sudanese refugees. While the original instrument defines scores of 8-12 as moderate distress, the adaptation to a lower threshold was considered appropriate for this population due to the specific context and heightened vulnerability observed in South Sudanese refugees. We acknowledge the need to further clarify this reasoning and provide additional citations to support this adjustment:*

*Moderate psychological distress was classified as greater than or equal to a K6 total score of 5 (*≥5) as in previous studies with South Sudanese refugees *(16,17) and others (18,19).* (page 8, line 164 – 166)

**R1.11** Line 190: provide examples of task shifting. What does that entail and how could this potentially impact experiences across participants?

***Response:*** *Thank you for the feedback. We have added the folowing reflections in the* ***methods****:*

*In this study, task shifting involved training non-specialists to deliver SH+ and ASSIST-BI to participants. This approach increases access to mental health services in resource-limited settings. The impact on participants' experiences can vary; some may feel more comfortable with familiar, community-based facilitators, while others may perceive non-specialist care as less formal or authoritative.* (page 9, line 192 – 196)

**R1.12** Line 197-199: Was the combined intervention validated on the study's population at any point during this study? If this study tries to fill the gap in knowledge by bringing interventions targeting two related but separate outcomes, these might need to be for future research directions or so.

***Response:*** *Thank you for your question. The combined intervention was validated on the study's population, and the findings related to this validation are reported in another article that is currently in the process of publication. This study aimed to assess the feasibility and acceptability of the combined intervention in this specific context, and the validation results will provide further insights into its effectiveness and applicability. Future research can build on these findings to explore further directions and potential adaptations for other populations or settings.*

**R1.13** Line 227: What kind of adaptations to the materials were made based on the feedback collected from intervention facilitators?

***Response:*** *Adaptations included refining images for the flip book and handouts that were more understable to the participants, and including context specific exercises and examples.*

Results
**R1.15** Table 1 is a bit confusing, consider reformatting. For example, why is the percentage of participants in each intervention group in the context of total participants if there were only 18 participants and the rest of the sample were family/facilitators?

***Response:*** *Thank you for the feedback. We have revised the table as suggested (page 14). For process evaluation, it was important to understand perceptions across people who had participated in ST+ and ASSIST-BI, family members and intervention facilitators. Thus, these are reffered to as “participants” of process evaluation interviews. We have added text to clarify this:*

*The participants of this study are categorised into three; (i) intervention participants, (ii) family members of intervention participants, and (iii) intervention facilitators.* (page 13, line 276 – 278)

**R1.16** The section on acceptability of the intervention doesn’t reference relevant content based on the earlier definition of acceptability referring to “satisfaction with the intervention components, content, delivery” (Line 114-115). The content in this section seems more aligned with the author's definition of relevance (Line 113-114), “the appropriateness, practicability and perceived usefulness of the intervention”.

***Response:*** *Thank you for the feedback. The section on the acceptability of the intervention was designed to capture participants' overall satisfaction with the intervention through their experiences of community building, social acceptance, and the sense of togetherness that emerged as a result of participating in the program. While these elements align with community-related outcomes, we believe they are critical to the overall acceptability of the intervention because they contribute to participants' perception of satisfaction with the content and delivery of the intervention. Specifically, sharing problems, socializing with people, and knowledge sharing directly relate to how participants experienced the content and delivery in a group setting, which enhanced their engagement and satisfaction with the intervention's social components. These elements of togetherness and relationships support the idea that participants felt more integrated into their community, which increased their satisfaction and engagement with the intervention—key components of acceptability. However, we acknowledge that these themes also touch on aspects of relevance, and we have also added our reflections on this:*

*While some of the themes, such as* ***community building****,* ***relationships****, and* ***social acceptance****, could be interpreted as aspects of* ***relevance****, these elements were integral to participants’ satisfaction with the social aspects of the intervention. Therefore, they were categorized under* ***acceptability*** *as they significantly influenced participants' overall experience and satisfaction with both the content and delivery of the intervention. (page 15, line 298 – 303)*

**R1.17** Line 559-563: mentioned other stakeholders and the importance of those relationships but who are the stakeholders and what was their role in this study? How did these individuals ease the mobilization of participants? Were there any additional impacts?

***Response:*** *Thank you for the feedback. The stakeholders highlighted by intervention facilitators are community leaders and church leaders (page 29, line 572):*

*This helped in the easier mobilisation of participants and also built trust among stakeholders as their community leaders communicated about the interventions prior to implementation.* (page 29, line 572 – 574)

Discussion
**R1.18** Line 693-694: It was indicated that more participants from the combined intervention participated in the process evaluation (over 40%). This should be noted earlier and more clearly in the results so readers can consider that when reviewing the main outcomes.

***Response:*** *Thank you for your feedback.* *We have revised the manuscript to ensure that the participation rate from the combined intervention group is mentioned earlier in the results section.*

**R1.19** New concepts such as livelihood interventions (lines 638-650) and gender roles (lines 652-671) are introduced at the end and while they are interesting they don’t seem to be directly related to the original study goals. Consider integrating this literature earlier on in the background section, particularly the content on gender norms.

***Response:*** *Thank you for your feedback. We have revised the manuscript to better integrate the discussions on gender roles into the introduction section:*

*Traditionally, South Sudanese men are viewed as family providers and protectors, but unemployment and reliance on humanitarian aid have disrupted these roles, leading to a loss of identity, heightened psychological distress, and increased substance misuse (18). The cultural expectation of masculinity intensifies these struggles, as many men face the burden of being unable to fulfill their traditional roles (18).* (page 5, line 91 – 96)
